# Effects of Spatial and Feature Attention on Disparity-Rendered Structure-From-Motion Stimuli in the Human Visual Cortex

**DOI:** 10.1371/journal.pone.0100074

**Published:** 2014-06-17

**Authors:** Ifan Betina Ip, Holly Bridge, Andrew J. Parker

**Affiliations:** 1 Department of Physiology, Anatomy & Genetics, University of Oxford, Oxford, United Kingdom; 2 Nuffield Department of Clinical Neurosciences, The Oxford Centre for Functional Magnetic Resonance Imaging of the Brain, University of Oxford, Oxford, United Kingdom; Ecole Normale Supérieure & CNRS, France

## Abstract

An important advance in the study of visual attention has been the identification of a non-spatial component of attention that enhances the response to similar features or objects across the visual field. Here we test whether this non-spatial component can co-select individual features that are perceptually bound into a coherent object. We combined human psychophysics and functional magnetic resonance imaging (fMRI) to demonstrate the ability to co-select individual features from perceptually coherent objects. Our study used binocular disparity and visual motion to define disparity structure-from-motion (dSFM) stimuli. Although the spatial attention system induced strong modulations of the fMRI response in visual regions, the non-spatial system’s ability to co-select features of the dSFM stimulus was less pronounced and variable across subjects. Our results demonstrate that feature and global feature attention effects are variable across participants, suggesting that the feature attention system may be limited in its ability to automatically select features within the attended object. Careful comparison of the task design suggests that even minor differences in the perceptual task may be critical in revealing the presence of global feature attention.

## Introduction

Allocation of attention can modulate the responses of neurons whose stimulus selectivity is similar to the attended feature [1], [2], [3]. The feature similarity gain model [4] summarizes these results: enhancement when attending to the preferred feature of a neuron and suppression for the opposite. Human functional magnetic resonance imaging (fMRI) studies suggest a ‘global feature attention’ effect in visual cortex, which enhances the response to similar features even when presented in different locations in the visual field [5], [6].

Direction of attention to specific features of a complex visual stimulus implies that observers can select out and respond to one stimulus component. This contrasts with cue combination, in which information from different features is brought together to form the most accurate estimate of the surface profile of the object. One example is the Bayesian combination of stereo and texture or luminance cues, in which the variance of the perceptual estimate of surface shape is reduced by exploiting information from more than one source [7], [8].

An even tighter link between cues is the use of one cue to resolve ambiguities in another [8]. An object’s depth structure can be perceived through relative motion cues alone (‘structure-from-motion’), yet the direction of rotation of an object rotating in depth is perceptually bistable [9], [10]. Binocular disparity and motion perceptually bind together, to the extent that disparity can disambiguate the depth position of ambiguous motion signals [11], [12], [13]. Psychophysical studies using adaptation also suggest that this linkage draws on shared neural representations. A disparity-contingent motion aftereffect has been demonstrated for disparity- and motion-defined stimuli: Adapting to a SFM-stimulus disambiguated by binocular disparity can bias the subsequent interpretation of an ambiguous stimulus to rotate in the opposite direction [13], [14]. Neurophysiological recordings in the macaque monkey have also shown that disparity and motion can be represented by neurons with joint selectivity to direction of motion and binocular disparity: binocular disparity resolved the depth ambiguity in SFM-stimuli, producing a consistent rotation [15], [16], [17].

Our experiment used dSFM-stimuli, which contain transparent moving regions of dots but appear perceptually as a single coherent cylindrical object rotating in depth [18]. If directed attention can co-select perceptually bound features of an object, then instructing participants to attend to specific features of the cylinder, such as its direction of rotation or speed, should modulate cortical responses to those features in a spatially adjacent, rotating cylinder. Therefore we used binocular disparity to manipulate the similarity between an attended and an unattended dSFM-stimulus. fMRI and human psychophysics were employed to test whether global feature attention could modulate responses within defined areas of the visual cortex.

## Methods

### Participants

Five participants (aged 23–27; 3 women; all right-handed) took part in the experiment. All had normal or corrected-to-normal vision and gave written informed consent in compliance with the guidelines of the Oxfordshire Research Ethics Committee A (06/Q1604/86). The Oxfordshire Research Ethics Committee ‘A’ provided ethical approval for this study. All passed the criterion for normal stereo-acuity at  = <120 arcsec disparity, as assessed by a clinical test of stereoscopic vision (TNO Test for Stereoscopic Vision, Lameris Instrumenten, Utrecht, The Netherlands). Each participant could correctly report the direction of rotation of a structure-from-motion cylinder disambiguated by ±0.09° center-to-front binocular disparity. Participants took part in a 1-hour session to obtain a T1-weighted structural image and retinotopic visual field maps and three separate 1-hour sessions to collect data for the main experiment. Three of the participants took part in additional control experiments.

### Experimental Paradigm

**Figure 1:**
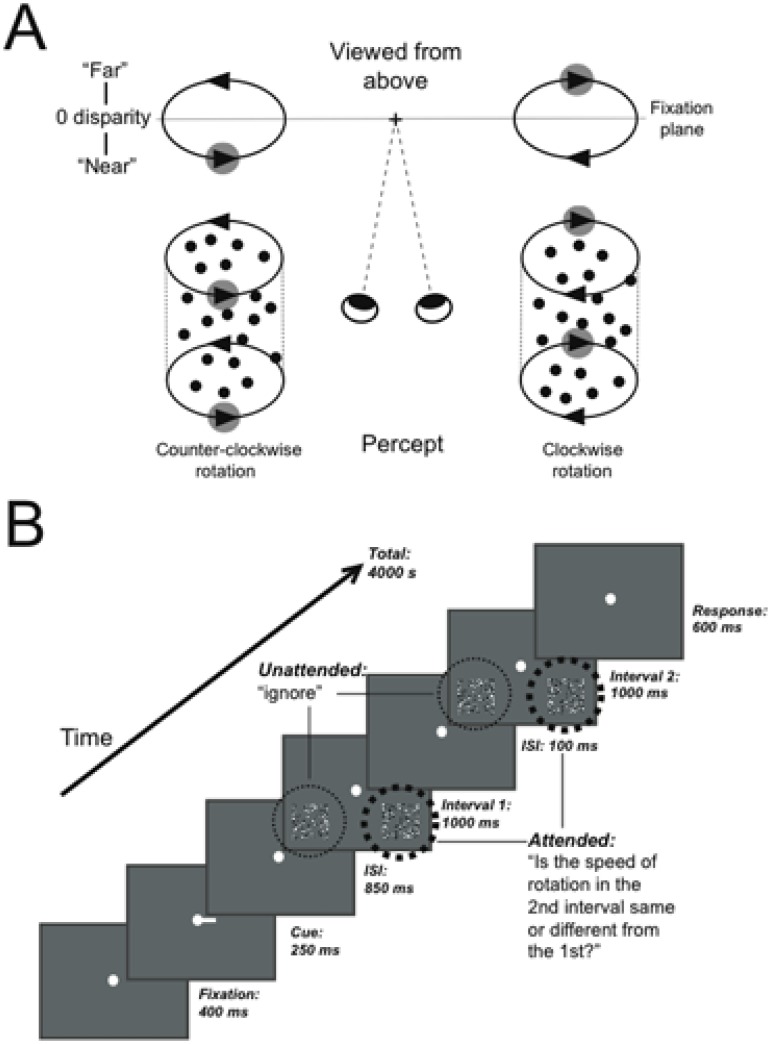
Stimuli and experimental paradigm. A: Structure-from-motion cylinders disambiguated by binocular disparity are perceived as rotating counter-clockwise (bottom left) or clockwise (bottom right) as controlled by the disparity of the right and left-wards moving surfaces (adapted from Dodd et al., 2001). B: Schematic diagram of the behavioral task used in the MRI-scanner, illustrating an example trial where attention is cued to the right side. For cued cylinders, participants reported whether the speed of rotation in the 1^st^ and 2^nd^ interval was different, while uncued cylinders were ignored.

Stimuli were structure-from-motion cylinders, each composed of a 5°×5° field of 125 white (57.5 cd/m^2^) and black (1.9 cd/m^2^) dots (0.2° size, anti-aliased for sub-pixel resolution) moving at a sinusoidal velocity and disparity profile in opposite directions around a vertical axis on a gray background (17.8 cd/m^2^). Cylinders were centered ±5.5° either side of the vertical midline and 4.5° below the horizontal fixation plane. The peak velocity and binocular disparity was at the midline of the cylinder and decreased towards the edges. A center-to-front disparity of 0.09° describes the absolute difference in degrees of visual angle between the nearest or farthest point of the cylinder to the fixation plane amounting to 0.09° positive and 0.09° negative disparity. A positive disparity corresponds to the rightwards surface moving in front of the fixation point and the leftwards surface behind. When viewed from the top, this generates the perception of counter-clockwise rotation. The reversed parameters generate clockwise rotation. Average angular rotation speed of cylinders ranged between 100°/s–177°/s, i.e. a dot would require between 2–3.6 s to complete a 360° rotation around the central axis. When a dot reached the edge of the cylinder, it was redrawn with the matching disparity and velocity gradient for dots moving in the opposite direction. Dots were plotted in randomly distributed positions: vertical positions were chosen homogenously along the height of the cylinder, and each dot position was slightly perturbed to prevent the appearance of a regular pattern of dots; horizontal positions were chosen to be homogenously distributed along 0–360° around the axis of the cylinder. The lifetime of individual dots was kept short to facilitate SFM-perception through motion and depth cues [10], [18], [19], [20]. On each video frame, 2% of the dots disappeared and were re-plotted in a random location on each video frame with a velocity appropriate to the new location. The dots of the cylinders were in new positions from one trial to the next. A schematic diagram of the experimental stimuli and the resulting perceptual interpretations are presented in Fig. 1A.

Fig. 1B shows a diagram of the experimental paradigm. A white fixation dot was always present and participants were instructed to maintain fixation on it at all times. In the example trial shown in Fig. 1B, a cue to the right indicated that the task-relevant cylinder would appear on the right side of fixation. On each trial, two intervals, each presenting a pair of cylinders, were shown and the task was to report whether the speed of the cylinder on the cued side was faster in the second interval than in the first. The thick dotted line indicates the attended side, the thin dotted line the unattended side. An equivalent sequence of events was presented for trials where attention was cued to the left. In a passive condition, which comprised the experimental baseline, trials were identical to the active condition trials except that stimuli were two fields of static, zero-disparity dots. The side on which the cue appeared in the passive condition was split 50/50 between left and right sides. The appearance of static dots signaled participants to passively fixate and alternately press the left-or right button in the response period. Randomly pressing buttons or guessing the speed-change would result in 0.5 proportion detected speed difference.

Participants were trained on the task in a psychophysical set-up prior to scanning (1295±243 average number of trials/participant). The speed change at which each participant could correctly determine the faster interval on 75% of trials was used for testing in the MRI-scanner. During training, the mean threshold across participants was a speed increase by a factor of 1.41±0.04 s.e.m compared with the original speed. At this value, participants would require a high level of focused attention to perform the task [21], [22] and subjects were considered to be performing the task incorrectly if scoring at 50% correct in the MRI-scanner, hence below 50% correct performance was used as a cut-off. All subjects performed significantly above 50% correct in the MRI-scanner (across subject mean = 0.68, 99% CI [0.66 0.70]). The unattended cylinder never changed speed between the 2-intervals in a trial. Instead, the unattended cylinder was set to rotate on different trials at randomly different speeds in comparison with the attended cylinder. This arrangement discouraged the use of comparisons between the two simultaneously visible cylinders to solve the speed discrimination task because the speeds of the unattended cylinder were not systematically related to those of the attended cylinder.

A block design was used because previously reported effects of global feature attention were small (∼0.05% of BOLD modulation [5]). Each scan presented 10 blocks (4 trials/block, each trial lasted 4 sec) of each of the six conditions. Blocks were pseudo-randomized and counterbalanced within scans. Within the scan, the unattended cylinder always rotated in the same direction, while the attended cylinder switched directions depending on the block. The direction of rotation of the unattended cylinder was alternated between scans.

To assess whether the experimental paradigm had succeeded in focusing attention on the speed task, a control experiment was devised that presented the same task with a valid cue (‘focused attention’- always pointing in the direction where a speed change occurred) or with an invalid cue (‘distributed’- sometimes pointing in the direction where a speed change occurred and sometimes in the opposite direction). If focused attention were used to perform the task, performance should be better in the focused compared to the distributed attention condition. Three participants from the main experiment took part in this control experiment. The average performance across participants for ‘valid’ cue type was significantly greater than for ‘invalid’ (valid: mean = 0.73, 99% CI [0.68 0.77]; invalid: mean = 0.53, 99% CI [0.46 0.59], corrected for multiple comparisons). This result confirms that the cue was successful at directing attention in the MRI-scanner.

### fMRI-set up and Psychophysics

Echo-planar images (EPI) (3×3×2.5 mm voxels, 45 slices, TR = 4 sec, TE = 30 ms, 192 mm FOV) were collected using a Siemens Trio 3T scanner (Oxford Centre for Magnetic Resonance, www.ocmr.ox.ac.uk/home) equipped with a 12-channel coil. A co-registered standard T1-weighted anatomical scan at twice the in-plane resolution (1.5×1.5×2.5 mm voxels, 44 slices, TR = 2350 ms, TE = 4 ms, 192 FOV) was collected on each session to aid registration. 240 volumes were collected per experimental scan and two scans were taken in each session. Hence, a total of 1440 volumes were collected per participant across three sessions. A high-resolution whole head T1-weighted structural scan (1×1×1 mm voxels, 192 slices, TR = 1 sec, TE = 4.7 ms, 192 FOV), optimized for gray-and white matter separation was collected once for each participant.

Binocular disparity in the MRI-scanner was generated using a CHRISTIE Mirage S+2K projector at 100 Hz and 1400×1050 pixel resolution. Binocular disparity was presented using alternate presentation of left and right eye images and polarized using a circularly polarizing Z-screen (RealID StereoGraphics, www.reald.com). The stereo-signal was quad-buffered with an OpenGL stereo system on a NVIDIA Quadro FX 1400 graphics controller and projected onto a back-projection screen that maintained the signal from the polarized light. Participants lay supine in the scanner, looked up into an angled mirror to the rear of the bore, and viewed the image through polarizing goggles. Soft wedges were used to stabilize head position and minimize head movement during scanning. The total viewing distance was 121 cm, the diameter of the scanner bore was 60 cm, yielding a visual field of 27.8°. Perceptual responses were given using the left-and right-most buttons of a MRI-compatible button box.

### Control for Stable Fixation

Fixation stability was monitored for two naive participants (Pt4, Pt5) wearing an MRI-compatible monocular eye-tracker (OberConsulting, Poland, 500 Hz, predicted spatial resolution of 0.5 arc degree) during collection of MRI-data for the main attention experiment (see Fig. S1). Eye position data from the MRI-scanner were analyzed using Matlab (The Mathworks, Natick, MA). Mean x (horizontal) and y (vertical) coordinates were measured for each trial and grouped by condition (baseline, attend right, attend left). Results showed no statistically significant effect of experimental condition on mean x or y-position (*t*-test, p>0.05). A limitation of calculating the averaged eye position is that mean position cannot reflect saccadic eye-movements, because a pro-saccade and return saccade would cause equal and opposite changes in the position vectors. Hence a pattern classification analysis using a linear support vector machine algorithm [23] was applied on trial-by-trial horizontal eye positions. A permutation test (10000 iterations of classification analysis) showed that the classifier could not significantly predict the side of attention or the direction of cylinder rotation from the eye positions significantly different from the null distribution. These results demonstrate that eye positions were not systematically related to experimental conditions.

### fMRI Data Analysis

fMRI data were analyzed using FSL 4.0 (http://fsl.fmrib.ox.ac.uk/fsl/fslwiki/), associated packages and scripts written in Matlab (The Mathworks, Natick, MA). For pre-processing, head movements were corrected using MCFLIRT [24] and the time series was high-pass filtered to remove low-frequency noise and slow drift [25]. The time courses were analyzed using a univariate linear model. Four conditions (attend left same/different, attend right same/different) were modeled as separate explanatory variables in the GLM-matrix. Regressors were convolved with the standard hemodynamic response function (gamma function with a delay of 6s, standard deviation of 3s) to account for the hemodynamic lag. Temporal derivatives, a slightly temporally shifted version of the model of each regressor, were added to account for latency differences between the modeled hemodynamic response and the data. Motion parameters were added as confound regressors to the statistical analysis to model out any changes in the signal that co-vary with residual head motion. The time series for each voxel was divided by its mean image intensity and the time series of voxels within each restricted regions-of-interest-mask was averaged. Statistical analysis on voxel time series was carried out using FILM with temporal autocorrelation correction [26]. Thresholded z-statistic maps consisted of clusters surviving statistical significance (p<0.01) and a cluster significance threshold of p<0.05 [27]. Functional and anatomical images were co-registered within participant [24], [28].

Regions-of-interest (ROI) were identified using a combination of retinotopic mapping and localizer scans. Firstly, visual areas were mapped in each participant following standard procedures [29], [30], [31], [32]. ROIs in the visual cortex (V1, V2, V3, V3a/b, hV4, V7, hMT+) were delineated in each hemisphere of the 5 participants using standard retinotopic mapping procedures and anatomical landmarks. The motion sensitive complex hMT+ was identified using the rotating wedge stimulus and anatomical landmarks, as a region located on the lateral occipital cortex, along the ascending limb of the inferior temporal sulcus [33], [34]. Secondly, a localizer scan was collected, presenting ambiguous SFM-cylinders in the same position as in the main experiment, alternating with a mid-gray screen with a fixation dot (blocks were 16 s on, 16 s off, 8 blocks/scan). As the cylinders for the localizer scans were perceptually ambiguous, responses were not biased towards either clockwise or counter-clockwise rotation. Participants were instructed to maintain passive fixation. Localizer scans were acquired within each session and averaged into a within-subject fixed-effects activation map. The resulting statistical map was used to restrict the number of voxels in each visual area to those significantly associated to the cylinder (z-stat >1.0, cluster threshold, p<0.05). A liberal threshold of z-stat >1.0 (equal to p<0.16) was used to ensure that thresholded visual areas were large enough to obtain responses. Cortical responses were quantified as the average change in %BOLD signal within an ROI.

Data were analyzed to assess effects of spatial attention and global feature attention. Comparing the amplitude of the BOLD signal when attention was directed towards a cylinder, to when attention was directed away from the same cylinder, identified effects of spatial attention. Effects of global feature attention were probed as the difference in BOLD response of the unattended cylinder when attention was directed to a cylinder rotating in the same direction compared to a cylinder rotating in an opposite direction. For each analysis, responses were calculated by averaging across individual participants’ scanning sessions, composed of averaged data from left and right hemispheres and 2 scans/session, yielding a total of 15 data points. Normality of data was assessed using the Kolmogorov-Smirnov test. Non-parametric Wilcoxon matched pairs tests were used to test for differences in median activation levels between conditions. Wilcoxon signed rank tests were used to assess response activation different from zero. Alpha levels were corrected for multiple comparisons (7 areas) using Bonferroni-correction.

### Retinotopy

Stimuli were displayed on a XGA projector (Sanyo, www.us.sanyo.com) on a rear-projection screen using a VSG 2/5 graphics card (Cambridge Research Systems, www.crsltd.com). Retinotopic data were collected using an EPI sequence (TE 30 ms; TR = 4000 ms; 2×2×2 mm resolution; FOV = 64×64) with a coronal orientation. To aid registration, an anatomical scan in the same orientation and with a resolution of 1×1×1 mm was acquired. Visual field eccentricity was mapped using an expanding concentric ring stimulus that traversed the visual field from the center towards the periphery. 32 volumes were acquired on each run, and one run lasted 192 s (moving outwards every 4 seconds, 4 seconds at each position, 8 positions, 6 cycles/scan, 32 s/cycle, diameter spanned by stimulus: 26°). The activation was estimated from the average of four runs. Visual field polar angle was mapped using a rotating wedge stimulus. Participants maintained passive fixation on a small red central fixation dot, while a wedge stimulus (45° wide, moving 30° every four seconds, 4 s at each position, 12 positions, 6 cycles/scan, diameter spanned by stimulus: 26°) traversed the visual field. The stimulus consisted of a wedge-shaped, white-and-black contrast reversing (8 Hz) checkerboard pattern. 72 volumes were acquired on each run, and one run lasted 288 s [35]. The activation maps were constructed from the average of four runs. For one participant, the polar angle of the visual field was mapped using a rotating wedge stimulus composed of coherent motion. 32 volumes were acquired on each run, and one run lasted 192 s. The stimulus consisted of a circle composed of 500 black dots on a white background. The motion was generated by dots that streamed alternately inwards and outwards, changing every second (90° wedge, moving 45° every four seconds, 4 seconds at each position, 8 positions, 6 cycles/scan) [35]. The activation map was estimated from the average of 7 runs. Data were processed using the mrVista package, the software can be obtained from http://vistalab.stanford.edu/newlm/index.php/MrVista [36]. A coherence map was obtained by a ratio that calculates the reliability of each voxel’s amplitude (signal) to a location in the visual field by dividing it by the summed amplitude (noise) of all remaining voxels. To expose areas buried in the sulci of the cortex and visualize the data, results were displayed onto a computationally inflated and flattened cortical surface [37].

### Multivariate Classification

Functional data were minimally pre-processed using MCFLIRT [24] motion correction and linear trend removal. Data were aligned to a functional reference volume. For each participant, a fixed-effects analysis was applied that isolated the most strongly activated voxels to dSFM-cylinders compared to static field of dots at zero-disparity (z-stat >2.3, cluster corrected). Using this statistical map, the 100 most activated voxels were identified for ROIs in the gray matter of the left and right hemispheres, and the time varying BOLD amplitude extracted. The functional time-courses were normalized (z-scored) to a mean of 0 and a standard deviation of 1. To remove univariate signals, the mean signal was projected out of each voxel separately. Time courses were shifted by 4 sec (1 volume) to account for the delayed peak in the hemodynamic response [38], [39]. Classifier labels were assigned depending on the trial type (attend left/attend right, rotate same/different, attended/unattended). One input ‘pattern’ was obtained by averaging across two trials that contained repetitions of a condition.

Multivariate pattern-classification for left and right ROIs separately (i.e. 100 voxels from left V1) was implemented using a linear support vector machine algorithm [23] with 5-fold hold-one-out cross validation and default parameters (C = 1.0). Cross-validation was achieved by splitting the data set into five sets. The classifier was trained on four sets and tested on the 5^th^ and the procedure reiterated until all five had been tested. Using this procedure, 120 patterns were made available for the feature attention classification (96 training, 24 testing patterns). Twice as many patterns were available for the spatial attention condition in comparison to the remaining conditions (192 training, 48 test patterns), because rotation of the unattended cylinder was irrelevant. The analysis was iterated 10000 times to obtain a stable prediction accuracy.

Significance of within and across subject classifier performance was tested using a non-parametric permutation test [40]. The null distribution was composed of 10000 iterations of the analysis with randomly shuffled condition labels using Matlab’s randperm. Classification accuracy parameters for the permutation were identical to the parameters used to obtain classification accuracies with correct label assignments.

## Results

### Behavioral Performance in the MRI-scanner

Participants performed the speed-difference task in the MRI-scanner. Average performance across participants was significantly above chance performance (mean = 0.68, 99% CI [0.66 0.7]). An N-way ANOVA with nested factors showed no statistically significant difference for behavioral performance across participants for main effects of similarity (same, different), stimulus condition (attend right same, attend right different, attend left same, attend left different), attended side (left, right), session (1–3) and global motion effects (rotation towards left or right side, inwards rotation, outwards rotation), p>0.05. These results confirm that behavioral performance in the MRI-scanner was not significantly different across stimulus conditions.

### Cortical Activity Maps of Spatial Attention

**Figure 2:**
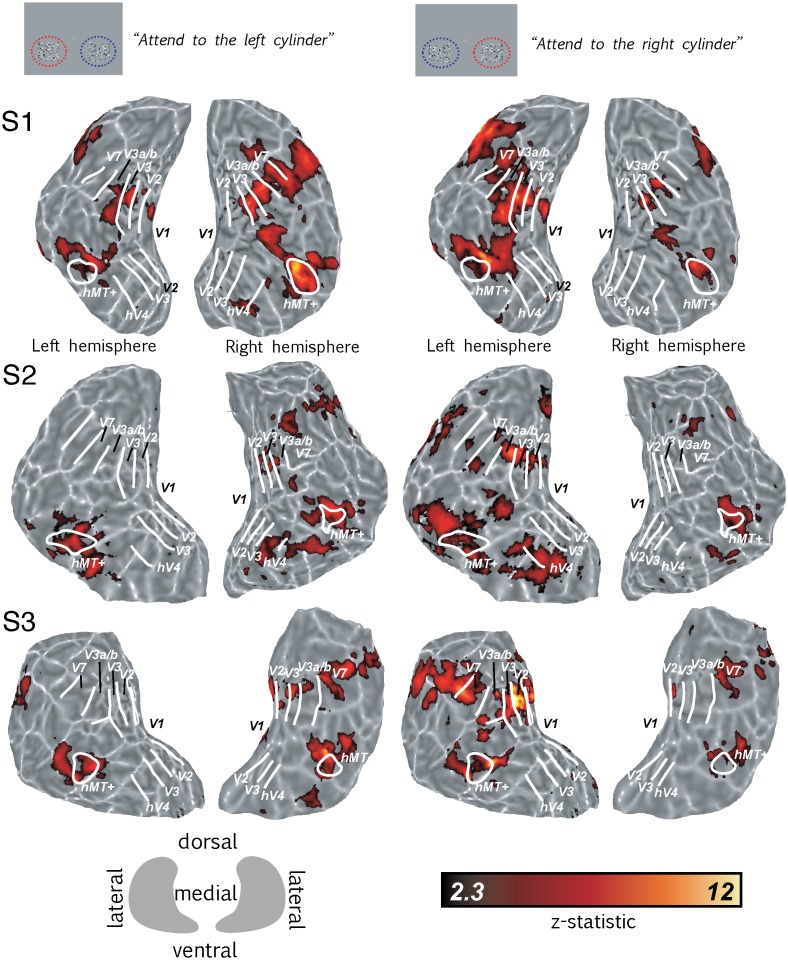
Spatial attention increased BOLD response to cylinders. Icons on the top show a schematic view of the stimulus screen from the perspective of the participant. Left half of figure shows responses to two cylinders compared to a baseline composed of two fields of static zero-disparity dots, with attention directed to the left cylinder. The right half of the figure shows the same conditions with attention directed to the right cylinder. Borders of visual areas (white lines) were defined using standard retinotopic mapping. All data were fully cluster-corrected at p<0.05. The color bar indicates significance levels of activation maps with a z-statistic ranging from 2.3–12. The key gives the orientation of the flat patch in relation to the dorsal, ventral and medial axis. Light gray areas mark gyri, dark gray areas sulci.

Significantly higher BOLD activity in the retinotopic visual cortex was generated by presenting two structure-from-motion cylinders disambiguated by binocular disparity (dSFM) compared to a baseline composed of two fields of static, zero-disparity dots. The focus of spatial attention was directed either to the left or to the right cylinder. Attention selectively increased BOLD responses in cortical regions associated with the attended cylinder. Fig. 2 left column, shows the cortical activation (compared to the baseline) when the left cylinder was attended and the right cylinder was ignored; Fig. 2 right column, shows the activation when the left cylinder was ignored and the right cylinder attended. Data are displayed on the flattened occipital lobes of single subjects with the statistical significance represented by color scale from red to yellow (high significance).

### Effects of Spatial Attention in Visual ROIs

**Figure 3:**
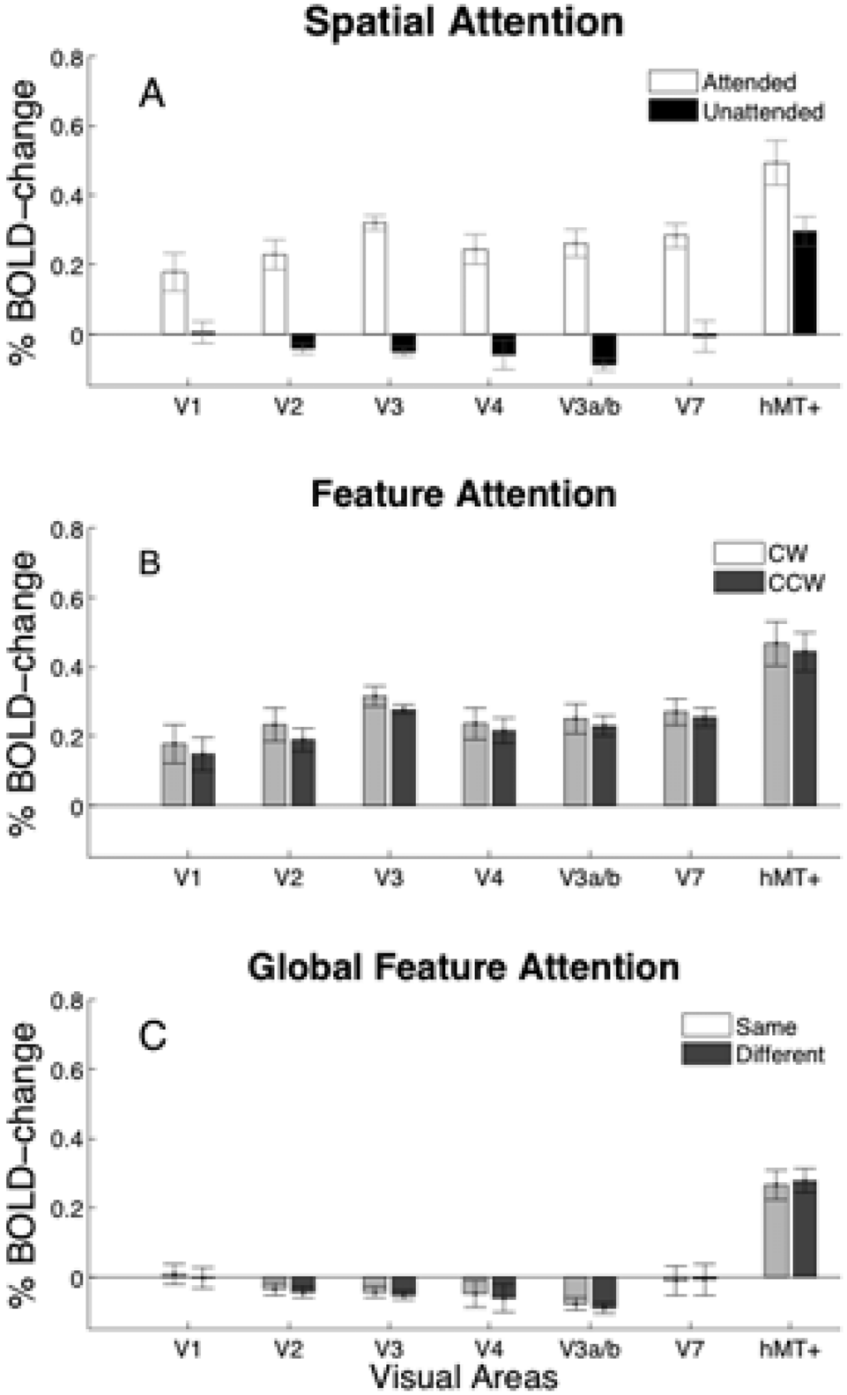
Cortical responses to cylinders. A: Cortical responses to cylinders disambiguated by disparity under attended (open) and unattended (filled) conditions compared to a baseline of static dots with zero-disparity. B: Average BOLD response to attended cylinders disambiguated by disparity under clockwise (light gray) and counter-clockwise (dark gray) conditions compared to a baseline of static dots with zero-disparity. C: Average BOLD response to unattended cylinder when rotating in the same (light gray) or different (dark gray) directions to the attended cylinder compared to the baseline. All errors are ± s.e.m. averaged across left and right hemispheres and two scans within participant sessions.

The 7 visual areas defined using retinotopy procedures (V1–V3, hV4, V3a/b, V7 and hMT+) were restricted to that part of the cortical area that was activated by a localizer scan which contrasted the response to two ambiguous SFM-cylinders compared to a blank gray screen. The responses to an attended or unattended cylinder compared to a baseline composed of a field of static zero-disparity dots are shown for each of these ROIs in Fig. 3A. A Wilcoxon signed rank test was used to assess areas that had activation greater than baseline. Attended cylinders generated significantly greater activity than baseline in all regions-of-interest. The response to unattended cylinders in V3 and V3a/b was significantly less than the uniform gray screen, whereas in hMT+ the response was significantly greater than baseline (p<0.05, corrected). Comparing attended to unattended activation, using a Wilcoxon matched pairs test, revealed significantly greater responses to the attended stimulus in V2, V3, hV4, V3a/b and V7 (p<0.001, corrected). There was no difference in cortical response between an attended cylinder rotating clockwise or counter-clockwise i.e. in the same or in the opposite direction from the unattended cylinder (Fig. 3B).

### Effects of Global Feature Attention in Visual ROIs

The feature similarity gain model predicts that attention will enhance the responses of sensory neurons tuned to the attended features across the entire visual field, with the magnitude of enhancement dependent on the similarity of the attended features to the sensory neuron under study [1], [2]. To reveal effects of global feature attention to dSFM-stimuli, the crucial comparison is the difference in BOLD activity to spatially unattended cylinders when they are rotating in the same, or different, direction as the attended cylinder. Importantly, the physical stimulus on the spatially unattended side does not change. Similarity is altered only by manipulation of the binocular disparity of the attended cylinder. Thus, any modulations on the unattended side would be due to the change in the similarity between the attended and unattended stimuli.

Fig. 3C shows the BOLD activity to unattended cylinders compared to a baseline of the activity to a field of static zero-disparity dots. A Wilcoxon matched pairs test showed that BOLD activity did not change as a function of attended cylinder rotation (p>0.05). A control analysis, using visual area masks that were not restricted by the independent localizer, also demonstrated significantly greater responses to the attended stimulus and no enhancement of responses due to the attended feature (see Fig. S2).

### Multivariate Pattern Analysis

The lack of an effect of global feature attention could potentially be due to insensitivity of the univariate analysis of fMRI data to spatially distributed information across an ROI. Univariate analysis measures the time course of the signal change averaged across a region-of-interest, so any weak but reliable signals that occur as differences between voxels would be lost. Multivariate pattern analysis examines this local spatial variation: it is sensitive to reliable, spatially distributed information across individual voxels in a region-of-interest and can be used to test whether patterns of activation to two (or more) conditions can be discriminated [38], [39]. Multivariate pattern analysis was used to test for global feature attention effects to dSFM-stimuli in the visual cortex. The discrimination provided by the analysis is expressed as proportion correct, where a value of 0.5 (chance) signifies that the pattern of activity within a particular visual area provides no discrimination between the two conditions and a value of 1.0 signifies that a perfect discrimination can be made.

**Figure 4:**
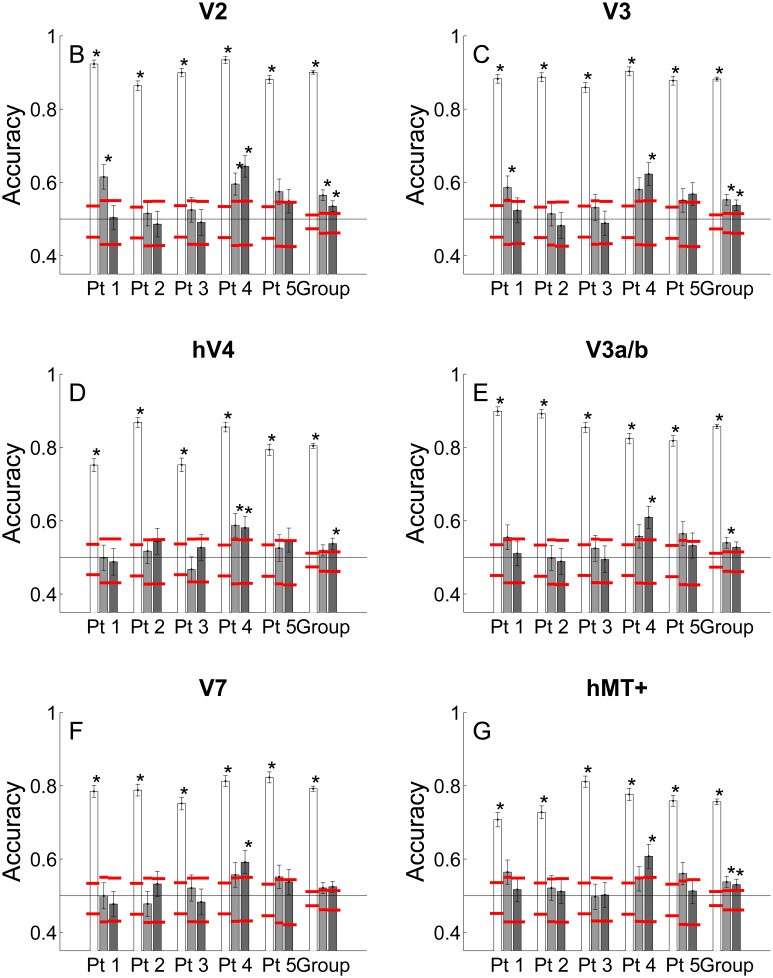
Mean classification accuracy to cylinders. A: Mean classification accuracy to a cylinder (in left or right visual field) in the 100 most activated voxels V1 and other retinotopic visual areas (B–G). The white bars show the classification based on attended or unattended condition. Light gray bars indicate the classification of an attended cylinder rotating in a clockwise or counter-clockwise direction. Dark gray bars show the classification of the unattended cylinder rotating in the same or different direction as the attended cylinder. The black line at 0.5 indicates chance performance. Black error bars indicate Bonferroni-corrected 95% confidence intervals obtained by iterating the classification 10000 times. Red lines indicate Bonferroni-corrected 95% confidence intervals of the empirical null distribution obtained by iterating the classification with permuted labels 10000 times. Asterices indicate significant classification accuracy.

Fig. 4 shows the mean classification accuracy for each region-of-interest, using the 100 voxels most activated by a single cylinder presented in the left or right visual field. To investigate the effects of spatial attention found in the univariate analysis, responses were first classified according to whether a cylinder in the left or right visual field was attended or unattended. As univariate signals have been explicitly removed in the pre-processing of the time courses by regressing out the mean signal prior to multivariate analysis, the classification should be driven by differences in the activity patterns between conditions. In agreement with the univariate results, several visual areas contained multivariate information, which reliably discriminated attended cylinders from unattended cylinders (Fig. 4, white bars).

To determine whether the direction of rotation of the attended cylinder could also be discriminated, classification was performed according to whether the cylinder was rotating clockwise or counter-clockwise (Fig. 4, light gray bars). For two of the five participants, classification rose above chance in early visual areas V1–V2 (p<0.05, non-parametric permutation test), confirming that attention co-selected the disparity defined rotation in depth of the attended stimuli.

To reveal effects of global feature attention, classification was performed on the responses to the unattended cylinder according to whether the unattended and attended cylinders were rotating in the same or different directions. Fig. 4, dark gray bars, shows that classification accuracy reached above chance in only one of the five participants, indicating that this participant’s responses contained reliable information for discriminating between cylinders rotating in the same or different directions. The group accuracies for feature attention and global feature attention comparisons were above chance in multiple visual areas (feature attention: V1, V2, V3, V3a/b, hMT+; global feature attention: V1, V2, V3, hV4, hMT+). This result for the group was actually due to above chance accuracies in one of the five participants.

## Discussion

Our study shows strong effects of spatial attention for dSFM-stimuli and, for a subset of participants, also attentional modulation of unattended dSFM-stimuli based on feature-similarity to attended dSFM-stimuli. A multivariate analysis showed that feature attention was present as a spatially distributed signal across voxels. Although multiple visual areas show significant accuracies for feature or global feature attention in the group results, single subject analyses showed that these were driven by up to two of the five participants. As expected, spatial attention increased BOLD activity to dSFM-stimuli across early and multiple ventral and dorsal visual areas. In summary, the results demonstrate that the feature similarity gain model is variable across participants when applied to stimuli defined by combined depth cues of structure-from-motion and binocular disparity. Our participants were carefully tested for their perceptual ability to register the differences in stimulus appearance brought about by manipulation of stereo disparity, so the failure to demonstrate feature-attention effects in some participants is not due to their insensitivity to stereo depth in these moving figures.

### Spatial Attention Modulates Multiple Visual Areas

Performing a behavioral task on dSFM-stimuli reliably increased BOLD activity in multiple visual areas, consistent with tight experimental control over the location of spatial attention. Visual areas V2, V3, hV4, V3a/b and V7 showed significantly greater BOLD activity when comparing responses to attended and unattended dSFM-stimuli. fMRI data has shown that ventral and dorsal visual areas located in the lateral-occipital regions are involved in processing of different types of disparity rendered shape [41], [42], [43], [44]. In agreement with these findings, we found that hMT+ was sensitive to dSFM-stimuli compared with the response to static fields of zero-disparity dots, even when attention was focused elsewhere. Furthermore, the multivariate classification analysis showed a statistically-significant classification-accuracy in V1, but this visual area did not show an effect of spatial attention in the univariate analysis. It is well established that sustained spatial attention can modulate cortical responses in V1 [45], [46] and extrastriate visual areas [47], [48], [49], even in absence of visual stimulation [45], [46], [50]. These modulations may also have driven fine scale differences in the fMRI voxel patterns, even after the univariate signal has been explicitly removed. Shifting spatial attention towards and away from dSFM-stimuli may have selectively changed responses of voxels with weak but reliable preferences for stimulus features. These preferences may reflect weak spatial clustering of disparity (V1, [51]; V2, [52], [53], [54]; MT, [15] or direction selective (MT, [55]) neurons in the visual cortex. Classification performance was consistent with the range of accuracies obtained by two recent MVPA-classification studies on human cortical responses to binocular disparity and motion-defined stimuli [43], [56]. Altogether, our results are in agreement with well-characterized effects of spatial attention on sensory responses in the human visual cortex [47], [48], [49], [50].

### No Consistent Effects of Global Feature Attention

Earlier fMRI studies of feature attention have studied transparent moving planes of dots, in which the planes are segregated perceptually [6], [57], [58] or using SFM-stimuli disambiguated by luminance cues [59]. The current experiment aimed to isolate global feature attention by systematically changing the similarity of rotation direction between attended and unattended SFM-stimuli using binocular disparity: no consistent modulation in activity based on similarity was present. Neurons in macaque V5/MT are highly sensitive to dSFM [16], [17] and such neurons could provide targets for attentional modulation of dSFM based on similarity across the visual field, an effect first shown in responses to stimuli defined by coherently moving dots in direction-selective neurons in V5/MT [1], [2]. However, the current findings do not support the feature similarity gain model, because a systematic change in similarity did not evoke a measurable change in the gain of neuronal populations in all participants.

Previous fMRI studies found greater activity in human visual cortex to an unattended stimulus when attention was directed to a coherently moving random dot surface streaming in the same direction as compared with the opposite direction [5], [60]. In those experiments, attention was directed to the task-relevant stimulus by asking participants to detect a change in the speed of the motion. Our paradigm also directed attention to the speed of the dSFM-stimulus. Moreover, our stimuli contained exactly the configuration of separate moving depth planes that led to early identification of a feature attention effect [5].

One possible explanation for the discrepancy is that the task-relevance of the speed of motion may have reduced the differences in activity to binocular disparity [61]. However, this seems unlikely, given that there are multiple studies demonstrating that global feature attention transfers to all features bound within an attended stimulus. A psychophysical correlate for the global feature attention effect, the motion-after-effect at remote locations, was present regardless of whether the motion or the color of the attended stimulus was task-relevant [62]. Melcher et al., showed that when the color of an unattended sub-threshold motion prime was matched to a coherently moving stimulus, the motion detection threshold at the unattended location was significantly decreased [63]. Finally, Katzner et al., showed that attending to the color or the motion of a single coherently moving stimulus evoked comparable global feature attention effects to motion-defined stimuli in area MT [64].

In the dSFM stimuli, motion and binocular disparity both define consistent cues for depth through sinusoidal velocity and disparity gradients. Psychophysical, computational and neurophysiological studies have shown that direction selectivity and disparity selectivity converge upon the same neural substrates [13], [14], [16]. These observations strongly suggest that when a stimulus is selected by spatial attention, all features belonging to an attended stimulus are subject to similar attentional modulation. Consistent with object-based attention theories [58], [65], attending to the motion of the dSFM-stimulus would have automatically selected the binocular disparity.

### Possible Role of Distracter as Surface Segregation Cue

Although superficially a dSFM-stimulus and coherently moving random dot stimuli with a superimposed distracter surface are very much alike, the critical difference in the dSFM stimulus may be that velocity and binocular disparity gradients bind surfaces moving in opposite directions into a single stimulus. In contrast, previous studies presented stimuli for which the attended and distracter surfaces segregated. These appeared like two distinct surfaces sliding across each other [5], [6], [66]. Such surface segregation cues generated by the distracter may switch on the binding of similar features into common surfaces [67], thus enabling object-based attention across to each of the perceptually segregated surfaces [58], [68]. If this were the case, stimuli bound into the same surface across the visual field would be facilitated, while stimuli that belong to a different surface would be suppressed.

It may be further noted that the psychophysical measures of feature attention conducted by Saenz, Buracas and Boynton [66] focus on testing just three subjects, two of whom were the authors of the study and presumably therefore aware of the hypotheses under test. The configuration that gave rise to the strongest psychophysical measures of global feature attention contained both target and distractor motions in both attended and non-attended stimuli. Stimulus configurations that contained only the target motion in the attended location and the opposite motion in the non-attended location showed no evidence of feature attention effects at the psychophysical level. Interestingly, the one configuration that has been extensively tested for fMRI responses in both this study and previous studies was intermediate between these two cases. The configuration studied for fMRI contains both target and distractor motions in the attended location but only one motion stimulus (either planar motion or direction of rotation of the cylinder) in the non-attended location [5]. Greater detail of specification for the stimulus and task may be needed to identify exactly the circumstances in which global feature attention emerges as a robust phenomenon.

## Conclusion

The feature similarity gain system may be limited in its ability to co-select features that together define a coherent object. The motion and binocular disparity features are tightly bound together because their joint encoding is essential for perceptually disambiguating the direction of rotation of the attended and unattended stimuli. Despite this tight link, feature attentional modulation was variable across participants as measured in terms of cortical activity. Our study suggests that the ability of the feature similarity gain system to co-select bound visual features may not be automatic. Instead, the presence of a measurable enhancement of cortical activity by feature attention may ultimately depend on the details of neural encoding of the features and the perceptual task that is performed.

## Supporting Information

Figure S1 **Mean eye position recordings.** Top row: Mean eye position from two naïve participants, for whom eye tracking data were concurrently recorded during MRI-data collection for the main experiment. Average *x* (open bar) and *y*-positions (black bar) when attention was cued to the left and to the right. Negative values stand for positions to the left, positive for positions to the right. Data were detrended to remove baseline drift. Note different *y*-axis for Pt4: calibration data were not available and data were z-normalized prior to analysis to obtain a mean of 0. Error bars show ± s.e.m. across individual trials. There was no significant difference in mean eye position between attention to the left and to the right for horizontal or vertical eye positions (*t*-test, p>0.05). Bottom row: Support vector machine classification with patterns composed of horizontal eye position data from individual trials. This analysis would have uncovered systematic changes due to saccadic eye-movements, which may have been averaged out in the analysis in the top row. Left plot shows classification results for Pt4 and right plot for Pt5 showing the mean and 95% confidence intervals for 10000 classification iterations. Data for analyses discriminating attend left vs right condition were pooled across same and different conditions. Analyses discriminating same vs different were applied to left or right attention conditions separately and then averaged. The solid black line at 0.5 proportion correct shows chance performance. Dashed lines show the 95% binomial confidence intervals of the null distribution generated by 10000 classification iterations with randomized condition labels.
(TIFF)

Figure S2 **Cortical responses measured using retinotopic visual areas that were not masked by the independent localizer.** A: Responses show cortical activity to cylinders disambiguated by disparity under attended (open) and unattended (filled) conditions compared to a baseline of static dots with zero-disparity. B: Average BOLD response to unattended cylinder when rotating in the same (light gray) or different (dark gray) directions to the attended cylinder compared to the baseline. All errors are ± s.e.m. averaged across left and right hemispheres and two scans within participant sessions. *indicates statistically significant comparison between attended and unattended responses.
(PNG)
