# Supplementary figures and images for: Effects of Spatial and Feature Attention on Disparity-Rendered Structure-From-Motion Stimuli in the Human Visual Cortex
Source: PLoS One. 2014 Jun 17;9(6):e100074. doi: 10.1371/journal.pone.0100074 (PMC4061053; doi:10.1371/journal.pone.0100074)

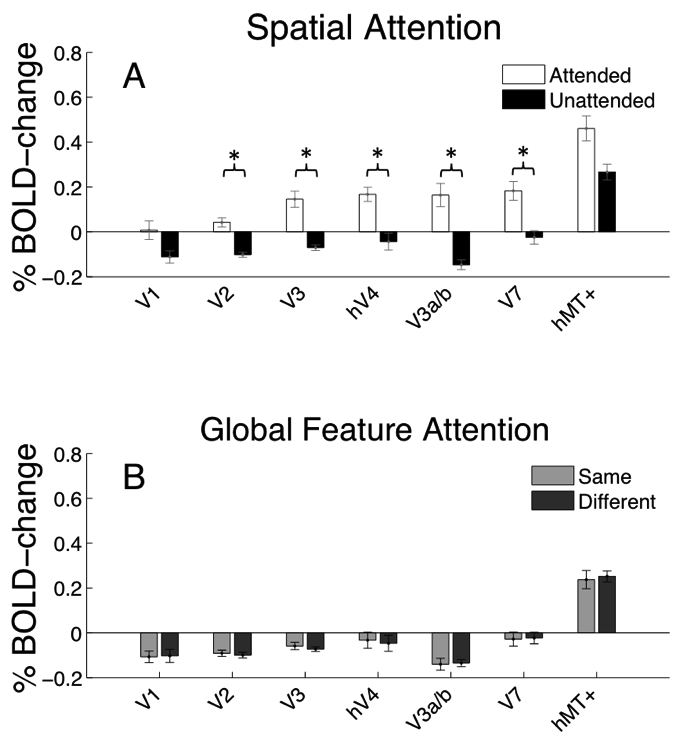

Supplement: Figure S2 — Cortical responses measured using retinotopic visual areas that were not masked by the independent localizer. A: Responses show cortical activity to cylinders disambiguated by disparity under attended (open) and unattended (filled) conditions compared to a baseline of static dots with zero-disparity. B: Average BOLD response to unattended cylinder when rotating in the same (light gray) or different (dark gray) directions to the attended cylinder compared to the baseline. All errors are ± s.e.m. averaged across left and right hemispheres and two scans within participant sessions. *indicates statistically significant comparison between attended and unattended responses. (PNG) [file pone.0100074.s002.png]
